# Supplementary material for: Observation of spin-current striction in a magnet
Source: Nat Commun. 2022 May 11;13:2440. doi: 10.1038/s41467-022-30115-y (PMC9095864; doi:10.1038/s41467-022-30115-y)
Supplement: Supplementary file 1 — Supplementary Information [file 41467_2022_30115_MOESM1_ESM.pdf]

Supplementary Information for

Observation of spin-current striction in a magnet

Hiroki Arisawa,[1,*] Hang Shim,[2] Shunsuke Daimon,[3,4] Takashi Kikkawa,[1,3,5] Yasuyuki Oikawa,[5] Saburo Takahashi,[5] Takahito Ono,[2,6] and Eiji Saitoh[1,3-5]

[1]*Institute for Materials Research, Tohoku University, Sendai 980-8577, Japan.*

[2] *Department of Mechanical Systems Engineering, Tohoku University, Sendai 980-8579, Japan.*

[3] *Department of Applied Physics, The University of Tokyo, Tokyo 113-8656, Japan.*

[4] *Institute for AI and Beyond, The University of Tokyo, Tokyo 113-8656, Japan.*

[5] *WPI, Advanced Institute for Materials Research, Tohoku University, Sendai 980-8577, Japan.*

[6] *Micro System Integration Center (μ-SiC), Tohoku University, Sendai 980-8579, Japan.*

Table of Contents

Supplementary Note 1 | Comparison of mechanical vibration between (Pt, W, and Cu)/$Tb_{0.3}Dy_{0.7}Fe_2$ and (Pt, W, and Cu)/Si samples.

Supplementary Note 2 | Temperature increase of sample surface due to Joule heating.

Supplementary Note 3 | A.c. current frequency dependence of mechanical displacement.

Supplementary Note 4 | Comparison between magnetic field dependence of mechanical signal and magnetization for Pt/$Tb_{0.3}Dy_{0.7}Fe_2$ sample.

Supplementary Note 5 | Sample preparation and sample properties.

Supplementary Note 6 | Magnetostriction measurement setup.

Supplementary Note 7 | Theoretical calculation of magnetization change induced by spin current injection.

Supplementary Note 8 | Phenomenological theory for mechanical displacement due to spin current injection via spin-lattice coupling.

Supplementary References

Supplementary Note 1 | Comparison of mechanical vibration between (Pt, W, and Cu)/$Tb_{0.3}Dy_{0.7}Fe_2$ and (Pt, W, and Cu)/Si samples.

We performed control experiments by comparing mechanical vibrations for the (Pt, W, and Cu)/$Tb_{0.3}Dy_{0.7}Fe_2$ and (Pt, W, and Cu)/Si samples. Supplementary Fig. 1a shows the frequency f spectrum of the signed vibration amplitude $d \equiv A\cos\phi$ for the Pt/$Tb_{0.3}Dy_{0.7}Fe_2$ (a blue solid curve) and Pt/Si samples (a black solid curve), where A and ϕ are the vibrational amplitude and phase, respectively. We applied the a.c. electric current $j_\mathrm{c} = 50$ mA with the frequency $f_\mathrm{AC} = 10$ kHz to the Pt film and the magnetic field $H = 1530$ Oe. A clear d peak appears at $f = 10$ kHz $(= f_\mathrm{AC})$ for the Pt/$Tb_{0.3}Dy_{0.7}Fe_2$ sample while $d \sim 0$ for the Pt/Si sample. The results exclude any mechanical effects in a Pt monolayer in response to j_c from possible origins of the observed mechanical vibration for the Pt/$Tb_{0.3}Dy_{0.7}Fe_2$ sample. We also confirmed that the d_peak, d at $f = f_\mathrm{AC}$, remains almost zero with changing H for the Si/Pt sample (black plots in Supplementary Fig. 1b). As shown in Supplementary Figs. 1c-1f, the mechanical signals for the W/Si and Cu/Si samples exhibit the same tendency as that for the Pt/Si sample. The results indicate that the Lorentz force due to an a.c. current in magnetic fields cannot explain the paramagnetic metal dependence of d_peak for the (Pt, W, and Cu)/$Tb_{0.3}Dy_{0.7}Fe_2$ samples. We also concluded that the influence of electrostriction of the paramagnetic metals is negligibly small. It is known that tungsten undergoes oxidation due to aging and may change the sign of the spin Hall effect. One of our tungsten samples was found to exhibit an inverted sign when measured about 20 months later.

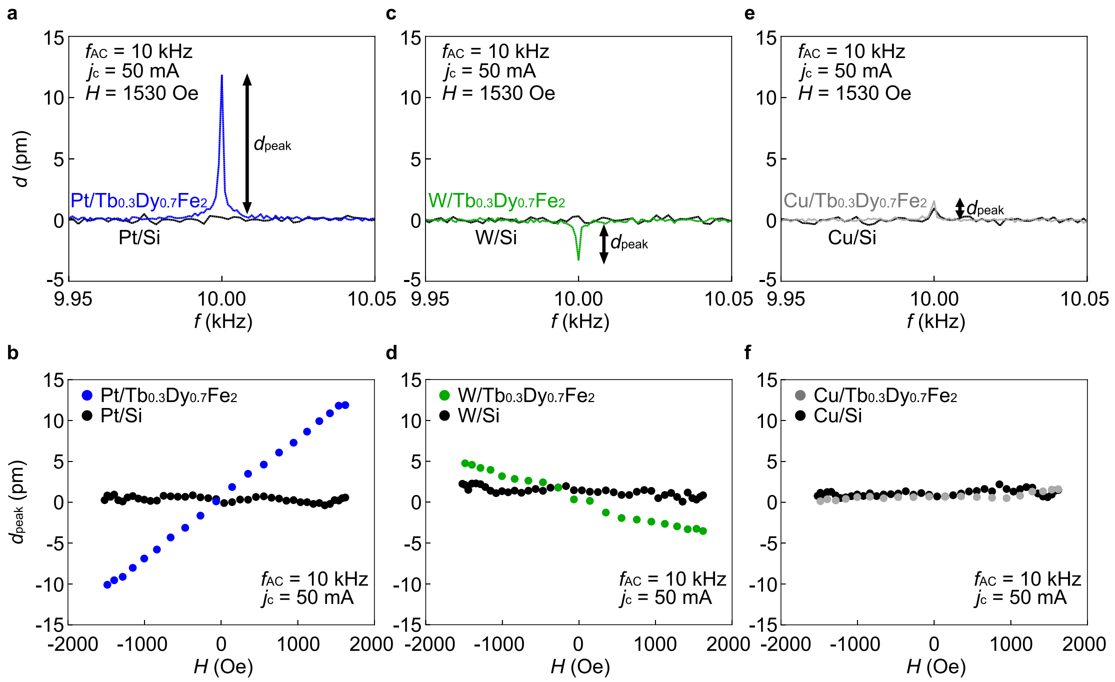

Supplementary Fig. 1 Mechanical vibration for (Pt, W, and Cu)/$Tb_{0.3}Dy_{0.7}Fe_2$ and (Pt, W, and Cu)/Si samples. a, c, e Frequency f spectra of the signed vibration amplitude d for the Pt/$Tb_{0.3}Dy_{0.7}Fe_2$ (a blue solid curve) and Pt/Si (a black solid curve) samples (**a**), the W/$Tb_{0.3}Dy_{0.7}Fe_2$ (a green solid curve) and W/Si (a black solid curve) samples (**c**), and the Cu/$Tb_{0.3}Dy_{0.7}Fe_2$ (a grey solid curve) and Cu/Si (a black solid curve) samples (**e**). The values of the a.c. current amplitude j_c, the a.c. current frequency f_{AC}, and the magnetic field H were set to 50 mA, 10 kHz, and 1530 Oe, respectively. **b, d, f** The H dependence of d_{peak}, d at $f = f_{AC}$, for the Pt/$Tb_{0.3}Dy_{0.7}Fe_2$ (blue plots) and Pt/Si (black plots) samples (**b**), the W/$Tb_{0.3}Dy_{0.7}Fe_2$ (green plots) and W/Si (black plots) samples (**d**), and the Cu/$Tb_{0.3}Dy_{0.7}Fe_2$ (grey plots) and Cu/Si (black plots) samples (**f**).

Supplementary Note 2 | Temperature increase of sample surface due to Joule heating.

We examined the influence of the Joule heating due to the a.c. current by measuring the sample temperature with a thermographic camera. Supplementary Fig. 2 shows the j_c dependence of the temperature increase ΔT for the Pt/$Tb_{0.3}Dy_{0.7}Fe_2$ sample surface. The value of f_{AC} was set to 10 kHz. Here, ΔT increases ($\propto j_c^2$) with the increase of j_c, and we confirmed that $\Delta T \sim 0.15$ K at $j_c = 50$ mA, which is much smaller than the system temperature in our experiment ~300 K. We also note that, around room temperature, the magneto-volume change of $Tb_{0.3}Dy_{0.7}Fe_2$ does not strongly depend on temperature [1]. From the result, we concluded that the influence of the Joule heating on the observed mechanical signal is negligibly small.

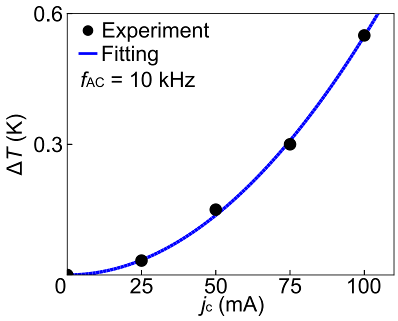

Supplementary Fig. 2 A.c. current dependence of sample surface temperature. The j_c dependence of the temperature increase ΔT of the Pt/$Tb_{0.3}Dy_{0.7}Fe_2$ sample surface (black dots). A blue solid curve is a square fitting. The value of f_{AC} was set to 10 kHz.

Supplementary Note 3 | A.c. current frequency dependence of mechanical displacement.

Supplementary Fig. 3 shows the f spectra of d for the Pt/$Tb_{0.3}Dy_{0.7}Fe_2$ sample at different f_{AC} values. We confirmed that a clear d peak appears at $f = 4, 5, 6, 7, 8, 9,$ and 10 kHz ($= f_{AC}$), exhibiting the frequency shift in response to the f_{AC} change. The values of j_c and H were set to 50 mA and 1530 Oe, respectively. The result indicates that the observed mechanical signal originates from the input a.c. current in the Pt film.

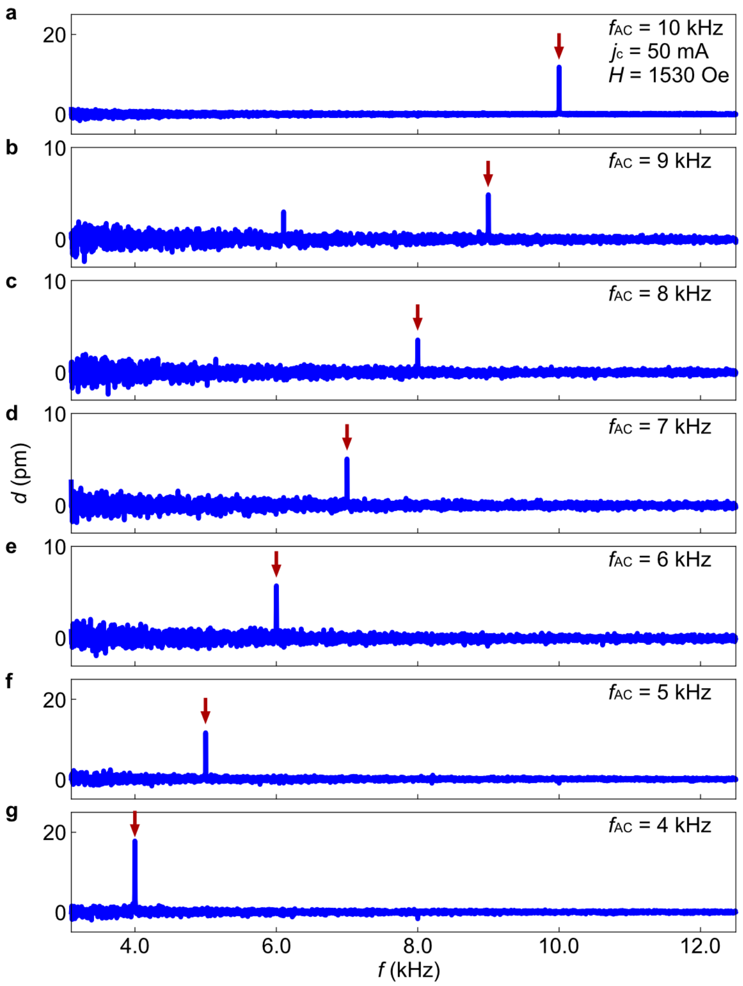

Supplementary Fig. 3 f spectra of d for Pt/$Tb_{0.3}Dy_{0.7}Fe_2$ sample at different f_{AC}. The values of j_c and H are 50 mA and 1530 Oe, respectively. The value of f_{AC} was set to 10 kHz (**a**), 9 kHz (**b**), 8 kHz (**c**), 7 kHz (**d**), 6 kHz (**e**), 5 kHz (**f**), and 4 kHz (**g**) at each measurement.

Supplementary Note 4 | Comparison between magnetic field dependence of mechanical signal and magnetization for Pt/$Tb_{0.3}Dy_{0.7}Fe_2$ sample.

In thin-film magnetism, the standard interpretation of an M-H curve that is nearly linear with small hysteresis is that the sample has a small amount of hard magnetic material (with hysteresis) scattered as impurities in a soft magnetic matrix phase with no hysteresis. According to this, the observed M-H curve can be interpreted as some impurity magnetic particles showing hysteresis scattered in the soft magnetic matrix phase of $Tb_{0.3}Dy_{0.7}Fe_2$, which exhibits large magneto-volume effect. The existence of this nominal impurity needs to be confirmed in the future from multiple angles, but it is consistent with the present experimental results, where the magneto-volume effect is considered to be dominated by the $Tb_{0.3}Dy_{0.7}Fe_2$ matrix phase.

Supplementary Note 5 | Sample preparation and sample properties.

We used an electroplating method [2] to grow the $Tb_{0.3}Dy_{0.7}Fe_2$ film on the paramagnetic metals. Supplementary Fig. 4a shows a schematic illustration of our electroplating system. We prepared solution containing Tb, Dy, and Fe ions, and placed a (Pt, W, and Cu)/Si substrate and a Pt wire in the solution as a working electrode and a counter electrode, respectively. To monitor the voltage between the two electrodes, we prepared a reference electrode, a AgCl wire, in KCl solution, which was electrically connected to the solution for the working and counter electrodes with a KCl salt bridge. By adjusting the voltage and deposition time at the electrolyte temperature 40 ℃, where the typical deposition rate is ~100 nm/hour, we fabricated the magnetic film with the thickness of ~100 nm on the Pt film with the thickness of 140 nm (the W and Cu films with the thickness of 100 nm).

We evaluated the chemical composition, magnetization, and magnetostriction of the magnetic film fabricated by the electroplating method. As shown in Supplementary Figs. 4d-4f, we carried out X-ray photoelectron spectroscopy (XPS) for the magnetic film fabricated on the Pt film and estimated the atomic composition ratio of the film as Tb:Dy:Fe ~11.1:22.5:66.4. From the result, the chemical composition of the film is given by ~ $Tb_{0.33}Dy_{0.68}Fe_{2.0}$, which is close to the optimal composition for large magnetostriction, $Tb_{0.3}Dy_{0.7}Fe_2$ [3]. By measuring the XPS spectra for the magnetic film fabricated on the W and Cu films (see Supplementary Figs. 4g-4i and 4j-4l, respectively), we confirmed the similar composition ~W/$Tb_{0.32}Dy_{0.74}Fe_{1.9}$ and ~Cu/$Tb_{0.29}Dy_{0.73}Fe_{2.0}$. As shown in Supplementary Figs. 4b and 4c, we also measured the H dependence of M and magnetostriction coefficient for the magnetic film [2] and found that the film exhibits magnetostriction coefficient $\sim 1200 \times 10^{-6}$ around the magnetization saturation, which is comparable to that of the bulk $Tb_{0.3}Dy_{0.7}Fe_2$ [3].

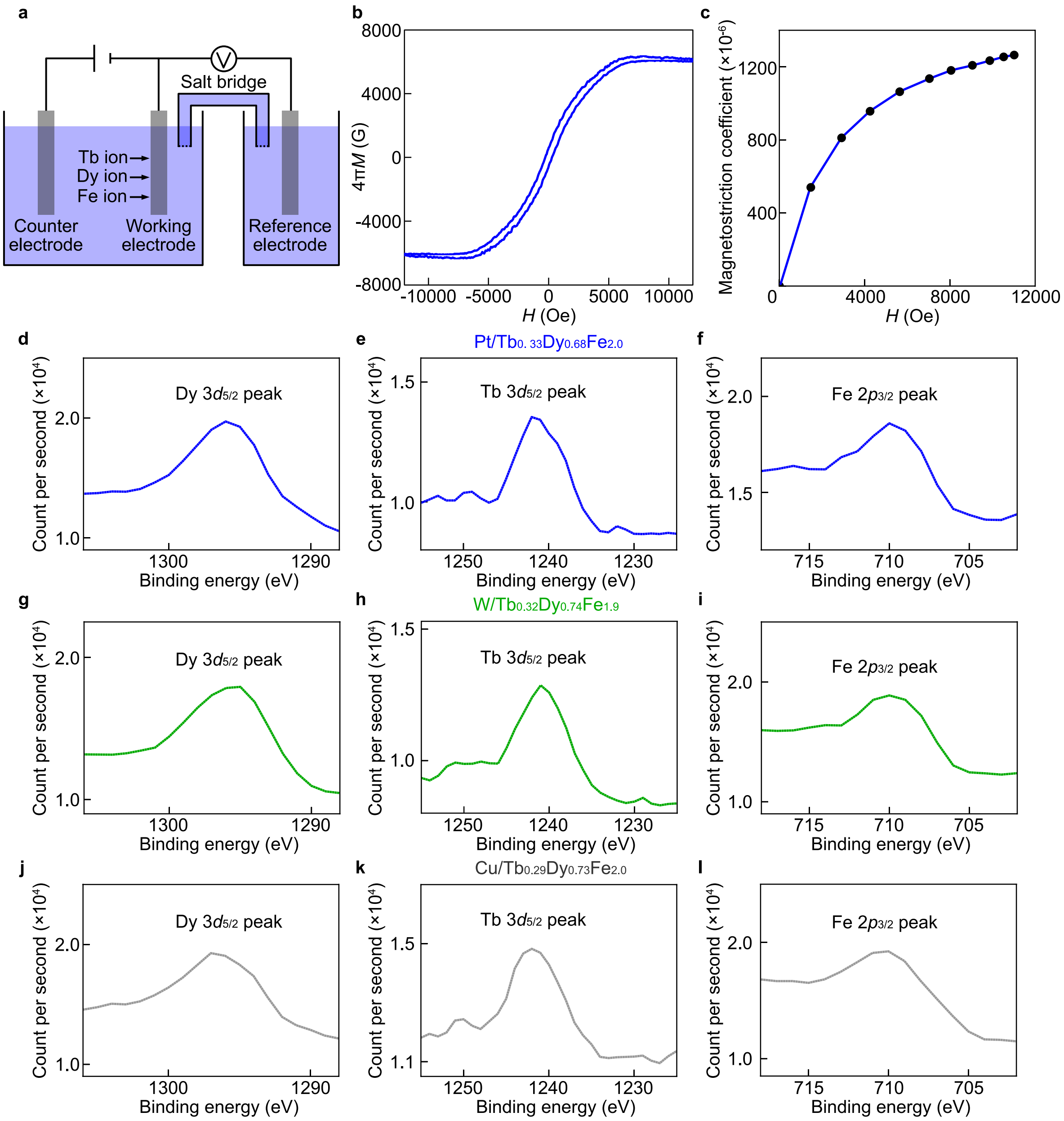

Supplementary Fig. 4 Sample fabrication and sample properties. a A schematic illustration of the electroplating deposition of the $Tb_{0.3}Dy_{0.7}Fe_2$ film. **b, c** The *H* dependence of *M* (**b**) and magnetostriction coefficient (**c**) for the magnetic film fabricated by the electroplating deposition [2]. **d, e, f** XPS spectra for the magnetic film on the Pt film. **g, h, i** XPS spectra for the magnetic film on the W film. **j, k, l** XPS spectra for the magnetic film on the Cu film.

Supplementary Note 6 | Magnetostriction measurement setup.

To evaluate magnetostriction of the $Tb_{0.3}Dy_{0.7}Fe_2$ film, we fabricated a tri-layer $Tb_{0.3}Dy_{0.7}Fe_2$/Cu/Si cantilever, whose size is typically 1 mm in length, 100 μm in width, and 2 μm in thickness, by chemical etching and measured the mechanical displacement of the cantilever in magnetic fields [2]. As shown in Supplementary Fig. 5, we applied H to the $Tb_{0.3}Dy_{0.7}Fe_2$/Cu/Si cantilever in the z direction and measured the H dependence of the mechanical displacement in the y direction by using an optical microscope. From the mechanical displacement of the cantilever in response to H, we calculated the H dependence of the magnetostriction coefficient of the $Tb_{0.3}Dy_{0.7}Fe_2$ film.

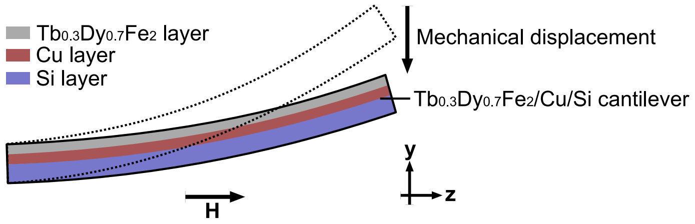

Supplementary Fig. 5 Schematic illustration of magnetostriction measurement of $Tb_{0.3}Dy_{0.7}Fe_2$ film.

Supplementary Note 7 | Theoretical calculation of magnetization change induced by spin current injection.

We calculate the spin fluctuation modulation due to spin current injection. It is convenient to consider the magnetization dynamics in the new xyz-coordinate system (Supplementary Fig. 6) in which we take the z-axis along the direction of magnetic field $\mathbf{H} = (0, 0, H)$ and define the relative angle between $\mathbf{H}$ and an electric current $\mathbf{j}_\mathrm{c}$ as θ. The spin current j_s with the spin polarization vector $\boldsymbol{\sigma} = (\cos\theta, 0, -\sin\theta)$ is injected from Pt into $Tb_{0.3}Dy_{0.7}Fe_2$. The magnetization dynamics in $Tb_{0.3}Dy_{0.7}Fe_2$ is described by the stochastic LLG equation[4]

$$\frac{d\mathbf{m}}{dt} = -\gamma\mathbf{m} \times [\mathbf{H} + \mathbf{h}(t)] + \frac{\alpha}{m_\mathrm{s}}\mathbf{m} \times \frac{d\mathbf{m}}{dt} + \frac{\gamma j_\mathrm{s}}{m_\mathrm{s}^2 s}\mathbf{m} \times (\mathbf{m} \times \boldsymbol{\sigma}), \tag{1}$$

where $\mathbf{m}$, γ, α, s, and m_s are the partial magnetization responsible for magnetostriction, the gyromagnetic ratio, the damping coefficient, the thickness of the $Tb_{0.3}Dy_{0.7}Fe_2$ film, and the saturation magnetization, respectively. Thermal fluctuation of $\mathbf{m}$ is taken into account by the random magnetic fields $\mathbf{h}(t) = (h_\mathrm{x}, h_\mathrm{y}, 0)$ satisfying $\langle h_i(t) h_j(t')\rangle = \frac{2\alpha k_\mathrm{B} T}{\gamma m_\mathrm{s} V}\delta_{i,j}\delta(t - t')$, where k_B, T, and V are Boltzmann constant, temperature, and the magnetic coherence volume[4] of the $Tb_{0.3}Dy_{0.7}Fe_2$ film, respectively.

We firstly calculate the steady state of the magnetization $\mathbf{m}^\mathrm{st} = (m_\mathrm{x}^\mathrm{st}, m_\mathrm{y}^\mathrm{st}, m_\mathrm{z}^\mathrm{st})$ without the magnetization fluctuation. By substituting $d\mathbf{m}^\mathrm{st}/dt = 0$ and $\mathbf{h}(t) = 0$ to Supplementary Eq. (1), we can obtain a solution

$$\mathbf{m}^\mathrm{st} \sim \left(0, -\frac{j_\mathrm{s}}{Hs}\cos\theta, m_\mathrm{s}\right), \tag{2}$$

up to the linear term with respect to j_s. This result means that the magnetization is rotated by the spin current injection to have an out-of-plane component in the y direction in the steady-state.

Next we take into account the magnetization fluctuation and calculate the fluctuation modulation due to the spin current injection. To extract the magnetization fluctuation component, we define the magnetization deviation from the steady state $\mathbf{m}^\mathrm{fluc} = \mathbf{m} - \mathbf{m}^\mathrm{st} = (m_\mathrm{x}^\mathrm{fluc}, m_\mathrm{y}^\mathrm{fluc}, m_\mathrm{z}^\mathrm{fluc})$. By substituting $\mathbf{m} = \mathbf{m}^\mathrm{st} + \mathbf{m}^\mathrm{fluc}$ to Supplementary Eq. (1) and linearizing the equation with respect to $\mathbf{m}^\mathrm{fluc}$ and $\mathbf{h}(t)$, we obtain

$$\frac{d\mathbf{m}^\mathrm{fluc}}{dt} = -\gamma\mathbf{m}^\mathrm{st} \times \mathbf{h}(t) - \gamma\mathbf{m}^\mathrm{fluc} \times \mathbf{H} + \frac{\alpha}{m_\mathrm{s}}\mathbf{m}^\mathrm{st} \times \frac{d\mathbf{m}^\mathrm{fluc}}{dt}$$

$$+ \frac{\gamma j_\mathrm{s}}{m_\mathrm{s}^2 s}\left[\mathbf{m}^\mathrm{st} \times \left(\mathbf{m}^\mathrm{fluc} \times \boldsymbol{\sigma}\right) + \mathbf{m}^\mathrm{fluc} \times (\mathbf{m}^\mathrm{st} \times \boldsymbol{\sigma})\right]. \tag{3}$$

With further using $m_\mathrm{x}^\mathrm{fluc} \gg m_\mathrm{z}^\mathrm{fluc}$, we can derive simultaneous differential equations of $m_\mathrm{x}^\mathrm{fluc}$ and $m_\mathrm{y}^\mathrm{fluc}$ as

$$\begin{pmatrix} \frac{d}{dt} + \frac{\gamma}{m_{\mathrm{s}}s} j_{\mathrm{s}} \sin\theta & \gamma H + \alpha \frac{d}{dt} \\ \gamma H + \alpha \frac{d}{dt} & -\frac{d}{dt} - \frac{\gamma}{m_{\mathrm{s}}s} j_{\mathrm{s}} \sin\theta \end{pmatrix} \begin{pmatrix} m_{\mathrm{x}}^{\mathrm{fluc}} \\ m_{\mathrm{y}}^{\mathrm{fluc}} \end{pmatrix} = \gamma m_{\mathrm{s}} \begin{pmatrix} 0 & 1 \\ 1 & 0 \end{pmatrix} \begin{pmatrix} h_{\mathrm{x}} \\ h_{\mathrm{y}} \end{pmatrix}, \tag{4}$$

Making use of the Fourier transformation, $\widetilde{m}_i^{\mathrm{fluc}} = \int_{-\infty}^{\infty} m_i^{\mathrm{fluc}}(t) \mathrm{e}^{-i\omega t} dt$ and $\tilde{h}_i = \int_{-\infty}^{\infty} h_i(t) \mathrm{e}^{-i\omega t} dt$, we obtain $\begin{pmatrix} \widetilde{m}_{\mathrm{x}}^{\mathrm{fluc}} \\ \widetilde{m}_{\mathrm{y}}^{\mathrm{fluc}} \end{pmatrix}$ in response to $\begin{pmatrix} \tilde{h}_{\mathrm{x}} \\ \tilde{h}_{\mathrm{y}} \end{pmatrix}$ as

$$\begin{pmatrix} \widetilde{m}_{\mathrm{x}}^{\mathrm{fluc}} \\ \widetilde{m}_{\mathrm{y}}^{\mathrm{fluc}} \end{pmatrix} = \begin{pmatrix} \tilde{\chi}_{\mathrm{xx}} & \tilde{\chi}_{\mathrm{xy}} \\ \tilde{\chi}_{\mathrm{yx}} & \tilde{\chi}_{\mathrm{yy}} \end{pmatrix} \begin{pmatrix} \tilde{h}_{\mathrm{x}} \\ \tilde{h}_{\mathrm{y}} \end{pmatrix}, \tag{5}$$

where the dynamical susceptibility is given by

$$\begin{pmatrix} \tilde{\chi}_{\mathrm{xx}} & \tilde{\chi}_{\mathrm{xy}} \\ \tilde{\chi}_{\mathrm{yx}} & \tilde{\chi}_{\mathrm{yy}} \end{pmatrix} = \frac{\gamma m_{\mathrm{s}}}{\left(-i\omega\alpha + \frac{\gamma j_{\mathrm{s}} \sin\theta}{m_{\mathrm{s}}s}\right)^2 + (\gamma H - i\omega\alpha)^2} \begin{pmatrix} \gamma H - i\omega\alpha & -i\omega + \frac{\gamma j_{\mathrm{s}} \sin\theta}{m_{\mathrm{s}}s} \\ i\omega - \frac{\gamma j_{\mathrm{s}} \sin\theta}{m_{\mathrm{s}}s} & \gamma H - i\omega\alpha \end{pmatrix}. \tag{6}$$

Now we can derive the expectation value of $(m_i^{\mathrm{fluc}})^2$ as

$$\left\langle (m_{\mathrm{x}}^{\mathrm{fluc}})^2 \right\rangle = \left\langle (m_{\mathrm{y}}^{\mathrm{fluc}})^2 \right\rangle = \frac{\alpha k_{\mathrm{B}} T}{\pi \gamma m_{\mathrm{s}} V} \int_{-\infty}^{\infty} \frac{(\tilde{\chi}_{\mathrm{xx}} \tilde{\chi}_{\mathrm{xx}}^* + \tilde{\chi}_{\mathrm{xy}} \tilde{\chi}_{\mathrm{xy}}^*)}{i\omega} d\omega \sim \frac{\alpha s m_{\mathrm{s}}^2 k_{\mathrm{B}} T}{V(\alpha s m_{\mathrm{s}} H + j_{\mathrm{s}} \sin\theta)}, \tag{7}$$

where we assumed that $\alpha \ll 1$. From the constraint condition of $\mathbf{m}^2 = m_{\mathrm{s}}^2$, $\langle \mathbf{m}^2 \rangle$ is given by

$$\langle \mathbf{m}^2 \rangle = \left\langle \left(m_{\mathrm{x}}^{\mathrm{st}} + m_{\mathrm{x}}^{\mathrm{fluc}}\right)^2 + \left(m_{\mathrm{y}}^{\mathrm{st}} + m_{\mathrm{y}}^{\mathrm{fluc}}\right)^2 + \left(m_{\mathrm{z}}^{\mathrm{st}} + m_{\mathrm{z}}^{\mathrm{fluc}}\right)^2 \right\rangle = m_{\mathrm{s}}^2. \tag{8}$$

By substituting Supplementary Eqs. (2) and (7), and $\langle m_{\mathrm{x}}^{\mathrm{fluc}} \rangle = \langle m_{\mathrm{y}}^{\mathrm{fluc}} \rangle = 0$ to Supplementary Eq. (8), we obtain

$$\left\langle (m_{\mathrm{z}}^{\mathrm{fluc}})^2 \right\rangle + 2 m_{\mathrm{s}} \langle m_{\mathrm{z}}^{\mathrm{fluc}} \rangle + \frac{2\alpha s m_{\mathrm{s}}^2 k_{\mathrm{B}} T}{V(\alpha s m_{\mathrm{s}} H + j_{\mathrm{s}} \sin\theta)} + \left(\frac{j_{\mathrm{s}}}{Hs} \cos\theta\right)^2 = 0. \tag{9}$$

Here, we use approximation $\left\langle (m_{\mathrm{z}}^{\mathrm{fluc}})^2 \right\rangle \sim \langle m_{\mathrm{z}}^{\mathrm{fluc}} \rangle^2$. This enables us to solve Supplementary Eq. (9) and obtain

$$\langle m_{\mathrm{z}}^{\mathrm{fluc}} \rangle \sim -\frac{k_{\mathrm{B}} T}{HV} + \frac{k_{\mathrm{B}} T}{\alpha s m_{\mathrm{s}} H^2 V} j_{\mathrm{s}} \sin\theta = -\frac{k_{\mathrm{B}} T}{HV} + \Delta m^{\mathrm{fluc}}. \tag{10}$$

Here, the first term is the magnetization decrease due to the thermal fluctuation of magnetization, and the second term $\Delta m^{\mathrm{fluc}} = \frac{k_{\mathrm{B}} T}{\alpha s m_{\mathrm{s}} H^2 V} j_{\mathrm{s}} \sin\theta$ originates from the magnetization fluctuation modulation via the spin current injection. From all these results, the final form of $\langle \mathbf{m} \rangle$ is given by

$$\langle \mathbf{m} \rangle = \mathbf{m}^{\mathrm{st}} + \langle \mathbf{m}^{\mathrm{fluc}} \rangle = \left(0, \Delta m^{\mathrm{st}}, m_{\mathrm{s}} - \frac{k_{\mathrm{B}} T}{HV} + \Delta m^{\mathrm{fluc}}\right), \tag{11}$$

where $\Delta m^{\mathrm{st}} = -\frac{j_s}{Hs}\cos\theta$ is the magnetization rotation component in the steady state.

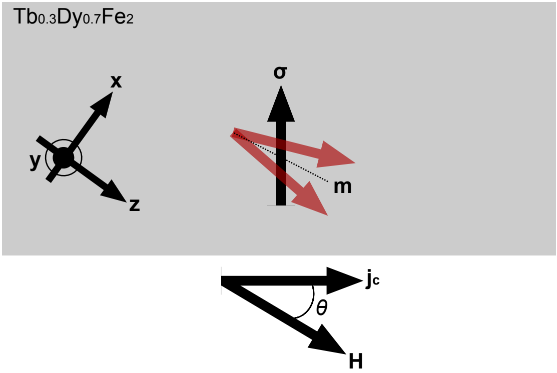

Supplementary Fig. 6 Schematic illustration of calculation setup. **H**, **m**, $\mathbf{j}_\mathrm{c}$, and **σ** denote the magnetic fields, the magnetization of the $Tb_{0.3}Dy_{0.7}Fe_2$ film responsible for magnetostriction, a charge current, and the spin polarization vector of the spin current, respectively. The z-axis is taken along the direction of **H**, and θ is the relative angle between **H** and $\mathbf{j}_\mathrm{c}$.

Supplementary Note 8 | Phenomenological theory for mechanical displacement due to spin current injection via spin-lattice coupling.

We calculate the mechanical displacement induced by the magnetization change in the $Tb_{0.3}Dy_{0.7}Fe_2$ film by using the phenomenological magnetoelastic model[5]. We define the Helmholtz free energy

$$F = F_{\mathrm{m}} + F_{\mathrm{el}} + F_{\mathrm{m-el}}, \tag{12}$$

$$F_{\mathrm{m}} = F_{\mathrm{m}}(m_i), \tag{13}$$

$$F_{\mathrm{el}} = \frac{c_{11}}{2}\left(e_{\mathrm{xx}}^2 + e_{\mathrm{yy}}^2 + e_{\mathrm{zz}}^2\right) + \frac{c_{44}}{2}\left(e_{\mathrm{xy}}^2 + e_{\mathrm{yz}}^2 + e_{\mathrm{zx}}^2\right) + c_{12}\left(e_{\mathrm{xx}}e_{\mathrm{yy}} + e_{\mathrm{yy}}e_{\mathrm{zz}} + e_{\mathrm{zz}}e_{\mathrm{xx}}\right), \tag{14}$$

$$F_{\mathrm{m-el}} = Q_{11}\left(e_{\mathrm{xx}}m_{\mathrm{x}}^2 + e_{\mathrm{yy}}m_{\mathrm{y}}^2 + e_{\mathrm{zz}}m_{\mathrm{z}}^2\right) + Q_{44}\left(e_{\mathrm{xy}}m_{\mathrm{x}}m_{\mathrm{y}} + e_{\mathrm{yz}}m_{\mathrm{y}}m_{\mathrm{z}} + e_{\mathrm{zx}}m_{\mathrm{z}}m_{\mathrm{x}}\right)$$
$$+Q_{12}\left[e_{\mathrm{xx}}\left(m_{\mathrm{y}}^2 + m_{\mathrm{z}}^2\right) + e_{\mathrm{yy}}\left(m_{\mathrm{z}}^2 + m_{\mathrm{x}}^2\right) + e_{\mathrm{zz}}\left(m_{\mathrm{x}}^2 + m_{\mathrm{y}}^2\right)\right], \tag{15}$$

where the valuables m_i and e_{ij} are the component of magnetization vector and the strain tensor, respectively, and c_{ij} and Q_{ij} are constant coefficients. By minimizing F with respect to e_{ij}, we obtain the magnetic strain as

$$e_{ii} = -\frac{c_{11}Q_{11} + c_{12}Q_{11} - 2c_{12}Q_{12}}{(c_{11} - c_{12})(c_{11} + 2c_{12})}m_i^2 - \frac{c_{11}Q_{12} - c_{12}Q_{11}}{(c_{11} - c_{12})(c_{11} + 2c_{12})}\left(m_{i+1}^2 + m_{i+2}^2\right), \tag{16}$$

$$e_{ij} = -\frac{Q_{44}}{c_{44}}m_i m_j \ (i \neq j). \tag{17}$$

The magnetic volume change component is given by

$$e_{\mathrm{xx}} + e_{\mathrm{yy}} + e_{\mathrm{zz}} = \frac{-Q_{11} - 2Q_{12}}{c_{11} + 2c_{12}}\left(m_{\mathrm{x}}^2 + m_{\mathrm{y}}^2 + m_{\mathrm{z}}^2\right). \tag{18}$$

By substituting Supplementary Eq. (11) to Supplementary Eq. (18), we can calculate the volume change when the spin current is injected to the $Tb_{0.3}Dy_{0.7}Fe_2$ film as

$$e_{\mathrm{xx}} + e_{\mathrm{yy}} + e_{\mathrm{zz}} \sim \frac{-Q_{11} - 2Q_{12}}{c_{11} + 2c_{12}}\left[\left(m_{\mathrm{s}} - \frac{k_{\mathrm{B}}T}{HV}\right)^2 + 2\left(m_{\mathrm{s}} - \frac{k_{\mathrm{B}}T}{HV}\right)\Delta m^{\mathrm{fluc}}\right], \tag{19}$$

where we made approximation up to the first order of j_{S}. The first term is the magnetic volume change due to the spontaneous magnetization and its thermal fluctuation. The second term corresponds to the volume change induced by the magnetization fluctuation modulation via the spin current injection. The out-of-plane displacement due to this volume change is given by

$$se_{\mathrm{yy}} = s\frac{c_{12}Q_{11} - c_{11}Q_{12}}{(c_{11} - c_{12})(c_{11} + 2c_{12})}\left(m_{\mathrm{s}} - \frac{k_{\mathrm{B}}T}{HV}\right)^2 + d_{\mathrm{SVE}}, \tag{20}$$

where d_{SVE} is the displacement due to the SVE as

$$d_{\mathrm{SVE}} = \frac{2k_{\mathrm{B}}T}{\alpha m_{\mathrm{s}} H^2 V} \frac{c_{12}Q_{11} - c_{11}Q_{12}}{(c_{11}-c_{12})(c_{11}+2c_{12})} \left(m_{\mathrm{s}} - \frac{k_{\mathrm{B}}T}{HV}\right) j_{\mathrm{s}} \sin\theta. \tag{21}$$

In addition to the volume change, the magnetization rotation term Δm^{st} causes the shear magnetostriction as

$$e_{\mathrm{yz}} \sim -\frac{Q_{44}}{c_{44}} \left(m_{\mathrm{s}} - \frac{k_{\mathrm{B}}T}{HV}\right) \Delta m^{\mathrm{st}} = \frac{1}{Hs} \frac{Q_{44}}{c_{44}} \left(m_{\mathrm{s}} - \frac{k_{\mathrm{B}}T}{HV}\right) j_{\mathrm{s}} \cos\theta, \tag{22}$$

resulting in the out-of-plane displacement $d_{\mathrm{ms}} \propto e_{\mathrm{yz}}$. From these results, we obtain the total out-of-plane displacement d_{tot} due to the spin current injection as

$$d_{\mathrm{tot}} = d_{\mathrm{SVE}} + d_{\mathrm{ms}}. \tag{23}$$

It is notable that

$$d_{\mathrm{SVE}} \propto j_{\mathrm{s}} \sin\theta, \qquad d_{\mathrm{ms}} \propto j_{\mathrm{s}} \cos\theta. \tag{24}$$

We quantitatively evaluate the displacement due to the spin current volume effect. We estimate d_{SVE} as $\sim s \frac{\lambda_{\mathrm{ms}}}{\Delta m} \Delta m^{\mathrm{fluc}}$, where Δm and λ_{ms} are the **m** change due to the field-induced **m** fluctuation change and magnetostriction in response to Δm, respectively. Here, we describe **H** as the sum of internal magnetic fields $\mathbf{H}_{\mathrm{int}}$ and the external magnetic fields $\mathbf{H}_{\mathrm{ext}}$. When $H_{\mathrm{int}} \gg H_{\mathrm{ext}}$, Δm is calculated as $\Delta m \sim \frac{\partial}{\partial H}\left(-\frac{k_{\mathrm{B}}T}{HV}\right)\Big|_{H=H_{\mathrm{int}}} \times H_{\mathrm{ext}} = \frac{H_{\mathrm{ext}} k_{\mathrm{B}} T}{H_{\mathrm{int}}^2 V}$. We also assume that λ_{ms} is linear with respect to H_{ext} ($\ll H_{\mathrm{int}}$), $\lambda_{\mathrm{ms}} = a_{\mathrm{ms}} H_{\mathrm{ext}}$, where a_{ms} is a constant parameter [2]. From the estimation we can obtain

$$d_{\mathrm{SVE}} \sim a_{\mathrm{ms}} \frac{j_{\mathrm{s}} \sin\theta}{\alpha m_{\mathrm{s}}} \sim a_{\mathrm{ms}} \left(\frac{\hbar}{2e} \frac{\theta_{\mathrm{SHE}} j_{\mathrm{c}}}{wu}\right) \frac{H_{\mathrm{ext}}}{\alpha m_{\mathrm{s}} H_{\mathrm{int}}}, \tag{25}$$

where θ_{SHE}, w, and u are the spin Hall angle, width, and thickness of the Pt film, respectively, and $\sin\theta$ is approximately replaced with $\frac{H_{\mathrm{ext}}}{H_{\mathrm{int}}}$. By fitting Supplementary Eq. (25) to the H_{ext} dependence of d_{peak} (blue plots in Fig. 3b in the main text), we obtained the unknown parameter $m_{\mathrm{s}} H_{\mathrm{int}}$ as in the order of $1\ \mathrm{J \cdot m^{-3}}$, which corresponds to the Zeeman energy for the partial magnetization of Tb and Dy ions in the internal field, responsible for magnetostriction of $Tb_{0.3}Dy_{0.7}Fe_2$ [3,6-8]. Here we used the parameters of $a_{\mathrm{ms}} = \frac{550 \times 10^{-6}}{1400}\ \mathrm{Oe^{-1}}$ [2], $\theta_{\mathrm{SHE}} = 0.113$ [9], $j_{\mathrm{c}} = 50 \times 10^{-3}$ A, $w = 2 \times 10^{-3}$ m, $u = 140 \times 10^{-9}$ m, and $\alpha = 0.06$ [10].

Supplementary References

1. Oomi, G. *et al.*, J. Magn. Soc. Japan **23**, 450-452 (1999).
2. Shim, H. *et al.* Magnetostrictive performance of electrodeposited $Tb_xDy_{1-x}Fe_y$ thin film with microcantilever structure. Micromachines **11**, 523 (2020).
3. Clark, A. E. in *Ferromagnetic Materials*, edited by E. M. Wohlfarth (North-Holland, Amsterdam, 1980), Vol. 1, p. 531.
4. Xiao, J., Bauer, G. E. W., Uchida, K., Saitoh, E. & Maekawa, S. Theory of magnon-driven spin Seebeck effect. Phys. Rev. B **81**, 214418 (2010).
5. Ishibashi, Y. & Iwata, M. A Theory of Morphotropic Phase Boundary in Solid-Solution Systems of Perovskite-Type Oxide Ferroelectrics. Jpn. J. Appl. Phys. **38**, 800-804 (1999).
6. Clark, A. E. Magnetic and Magnetoelastic Properties of Highly Magnetostrictive Rare Earth-Iron Laves Phase Compounds. AIP Conf. Proc. **18**, 1015 (1974).
7. Jiles, D. C. The development of highly magnetostrictive rare earth-iron alloys. J. Phys. D: Appl. Phys. **27**, 1 (1994).
8. Ren, W. J. and Zhang, Z. D. Progress in bulk $MgCu_2$-type rare-earth iron magnetostrictive compounds. Chin. Phys. B **22**, 077507 (2013).
9. Seki, T. *et al*. Giant spin Hall effect in perpendicularly spin-polarized FePt/Au devices. Nature Mater. **7**, 125–129 (2008).
10. Gopman, D. B., Lau, J. W., Mohanchandra, K. P., Wetzlar, K. & Carman, G. P. Determination of the exchange constant of $Tb_{0.3}Dy_{0.7}Fe_2$ by broadband ferromagnetic resonance spectroscopy. Phys. Rev. B **93**, 064425 (2016).
